# ^11^C-CFT PET brain imaging in Parkinson’s disease using a total-body PET/CT scanner

**DOI:** 10.1186/s40658-024-00640-4

**Published:** 2024-04-25

**Authors:** Xiaolin Sun, Xiaoyue Tan, Qing Zhang, Shanzhen He, Siyun Wang, Yongrong Zhou, Qi Huang, Lei Jiang

**Affiliations:** 1 PET Center, Department of Nuclear Medicine, Guangdong Provincial People’s Hospital, Guangdong Academy of Medical Sciences, Southern Medical University, 106 Zhongshan Er Road, 510080 Guangzhou, China; 2 Department of Nuclear Medicine & PET Center, Huashan Hospital, Fudan University, 518 Wuzhongdong Road, 200030 Shanghai, China; 3 Guangdong Provincial Key Laboratory of Artificial Intelligence in Medical Image Analysis and Application, Guangzhou, China

**Keywords:** Parkinson’s disease (PD), ^11^C-CFT, Total-body PET/CT, Scan time

## Abstract

**Purpose:**

This study aimed to evaluate the feasibility of ^11^C-CFT PET brain imaging in Parkinson’s Disease using a total-body PET/CT scanner and explore the optimal scan duration to guide the clinical practice.

**Methods:**

Thirty-two patients with Parkinson’s disease (PD) performing ^11^C-CFT PET/CT brain imaging using a total-body PET/CT scanner were retrospectively enrolled. The PET data acquired over a period of 900 s were reconstructed into groups of different durations: 900-s, 720-s, 600-s, 480-s, 300-s, 180-s, 120-s, 60-s, and 30-s (G900 to G30). The subjective image quality analysis was performed using 5-point scales. Semi-quantitative measurements were analyzed by SUVmean and dopamine transporter (DAT) binding of key brain regions implicated in PD, including the caudate nucleus and putamen. The full-time images (G900) were served as reference.

**Results:**

The overall G900, G720, and G600 image quality scores were 5.0 ± 0.0, 5.0 ± 0.0, and 4.9 ± 0.3 points, respectively, and there was no significant difference among these groups (*P* > 0.05). A significant decrease in these scores at durations shorter than 600 s was observed when compared to G900 images (*P* < 0.05). However, all G300 image quality was clinically acceptable (≥ 3 points). As the scan duration reduced, the SUVmean and DAT binding of caudate nucleus and putamen decreased progressively, while there were no statistically significant variations in the SUVmean of the background among the different groups. Moreover, the changes in the lesion DAT binding (ΔDAT-binding) between the full-time reference G900 image and other reconstructed group G720 to G30 images generally increased along with the reduced scan time.

**Conclusion:**

Sufficient image quality and lesion conspicuity could be achieved at 600-s scan duration for ^11^C-CFT PET brain imaging in PD assessment using a total-body PET/CT scanner, while the image quality of G300 was acceptable to meet clinical diagnosis, contributing to improve patient compliance and throughput of PET brain imaging.

## Introduction

Parkinson’s disease (PD), a prevalent neurodegenerative disorder, presents a significant and growing challenge in global healthcare. Characterized by the progressive degeneration of dopaminergic neurons in the substantia nigra, PD manifests in motor symptoms such as tremors, rigidity, and bradykinesia, as well as various non-motor symptoms [1, 2]. With the aging of the global population, the incidence of PD is projected to increase, underscoring the urgent need for advanced diagnostic and therapeutic approaches.

With advances in molecular neuroimaging, PET tracers targeting dopamine transporters (DAT) could reflect the functional integrity of dopaminergic neurons in the striatum [3], which has emerged as an important tool in the evaluation of PD [4]. For instance, several studies have demonstrated the utility of ^11^C-methyl-N-2β-carbomethoxy-3β-(4-fluorophenyl)-tropanel (^11^C-CFT) PET brain imaging in detecting the dopaminergic deficits characteristic of PD, thereby providing valuable insights into disease progression and aiding in differential diagnosis [5–9]. Currently, the proposed international consensus on ^11^C-CFT PET brain scan in parkinsonism recommends a 15–20 min scan duration [10]. Despite its potential, the clinical applications of ^11^C-CFT PET in PD face significant challenges, primarily regarding the relatively prolonged scan duration, which raises concerns about patient comfort and compliance, especially given the motor symptoms prevalent in PD patients. Furthermore, the extended durations can lead to a higher likelihood of movement-induced artifacts, potentially compromising the quality and reliability of the imaging data.

The emergence of the state-of-the-art PET/CT scanner with 194-cm-long axial field of view (FOV) and high detection efficiency provides an opportunity to further reduce acquisition duration or radiotracer activity [11, 12]. Several researches have employed various reconstruction algorithms on total-body PET/CT scanners to simulate low-count or rapid acquisition PET images [12, 13]. And previous studies have demonstrated that both the acquisition time and radiotracer activity can be substantially reduced, yet still preserve high diagnostic efficacy in oncological patients and have shown significant promise in pediatric patient populations [14–16]. However, there have been few studies exploring rapid acquisition of PET brain imaging using a total-body PET/CT scanner. In this study, we aim to investigate the reduction of possible acquisition time of ^11^C-CFT PET brain imaging that maintains sufficient image quality and diagnostic efficacy for PD assessment using total-body PET/CT.

## Methods

### Patients

Between May 2023 and October 2023, the consecutive patients performing ^11^C-CFT PET/CT brain scan using a total-body PET/CT scanner at Guangdong Provincial People’s Hospital were retrospectively analyzed. After excluding cases with poor image quality caused by severe motion effect or other factors, a total of 32 patients diagnosed with PD were finally enrolled in this study. The diagnosis of PD was established by clinical examinations by two specialists of movement disorders according to the UK Brain Bank criteria [17]. The clinical assessment for individual patient was evaluated using Hoehn and Yahr (H&Y) scale and was classified into different stages.

### Imaging protocol

^11^C-CFT PET brain imaging was acquired 60–80 min after an intravenous injection of ^11^C-CFT (350–550 MBq) using a total-body PET/CT scanner (uEXPLORER, United Imaging Healthcare, Shanghai, China) for 900 s (15 min) and reconstructed with the ordered subset expectation maximization (OSEM) method with 3 iterations and 20 subsets. Low-dose CT scans with tube current-time products 5 mAs before the static PET scan in a 3D mode were obtained and reconstructed for PET attenuation correction and diagnostic purposes. All patients were off anti-Parkinsonian medications for at least 12 h and rested in a quiet and dim room after the ^11^C-CFT injection prior to PET scans. The head of each patient was positioned in the middle of the total-body PET/CT scanner.

### Image reconstruction

The full-time raw PET data (acquisition time of 900 s) as well as the truncated data (720 s, 600 s, 480 s, 300 s, 180 s, 120 s, 60 s, and 30 s) were reconstructed using the OSEM algorithm incorporating time-of-flight and point-spread function modeling (TOF-PSF) on a medical image processing workstation (uWS-MI, United Imaging Healthcare). All PET/CT images were reconstructed with the following parameters: matrix of 256 × 256, slice thickness of 2.89 mm, FOV 300 mm with a Gaussian post-filter (4 mm). For the sake of simplicity, the image series reconstructed with 900 to 30 s were referred to as G900, G720, G600, G480, G300, G180, G120, G60, and G30, respectively.

### Subjective image quality assessment

The subjective PET image quality of the reconstructed series of PET datasets was independently evaluated by two experienced nuclear radiologists blinded to patient ID and the reconstructed acquisition time group. A 5-point Likert scale was used for (1) overall impression of the image quality, (2) image noise, and (3) conspicuity of lesions (basal ganglia with abnormal dopaminergic reduction). The visual scale for image quality consisted of score 5 to 1 that are defined as follows: (1) score 5: state-of-the-art quality, optimal signal-to-noise ratio, sharp lesion depiction; (2) score 4: superior to the regular image quality of daily practice; (3) score 3: equal to the regular quality of clinical routine image quality in our institution by a conventional PET/CT scanner; (4) score 2: barely diagnostic quality, or sub-optimal noise; and (5) score 1: non-diagnostic quality, excessive noise, or unfavorable lesion contrast.

### Semi-quantitative imaging analysis

^11^C-CFT PET brain data was analyzed using SPM12 software (Statistical Parametric Mapping; Wellcome Department of Imaging Neuroscience, Institute of Neu rology, London, UK) implemented in Matlab 2022b (MathWorks Inc., Sherborn, MA). Scans from each subject were spatially normalized into Montreal Neurological Institute (MNI) brain space with with a toolbox for spatial normalization of brain PET images (SNBPI) [18]. Then the SUVmean values for the bilateral caudate, anterior putamen, posterior putamen, and occipital cortex were extracted. The occipital cortex was selected as the background reference. DAT binding was estimated for each hemisphere by the striatal-to-occipital ratio, defined as (striatum-occipital)/occipital counts. Changes in DAT binding (ΔDAT-binding) of these regions were calculated by subtracting DAT binding of different duration groups from the G900 group.

### Statistical analysis

SPSS 26.0 software (IBM Corp., Armonk, NY, USA) was used for the statistical analysis. To overcome the difference in SUV among patients due to individual metabolism and disease severity. Related-Samples Wilcoxon Signed Rank Test was used for subjective image quality and semi-quantitative measurement analyses between different groups. *P* < 0.05 is considered statistically significant.

## Results

### Patient characteristics

This study included 32 patients (23 males and 9 females) diagnosed with PD who underwent ^11^C-CFT PET/CT brain imaging. The characteristics of these patients were summarized in Table 1. The average age of the patients was 60.7 years (± 12.9 SD), ranging from 30 to 87 years. The average disease duration of the patients was 22.9 months (± 17.1 SD). H&Y Stage distribution was as follows: stage 1, 56.3% (*n* = 18); stage 2, 28.1% (*n* = 9); and stage 3, 15.6% (*n* = 5). The mean injected dose of the tracer was 428.1 MBq (± 54.3 SD), with an average of 6.91 MBq/kg (± 1.58 SD) adjusted per weight. The waiting time between the tracer injection and PET brain scan averaged 70.9 min (± 9.4 SD).

**Table 1:** Clinical characteristics of the enrolled PD patients

Characteristics	Value
Age (years, mean ± SD)	60.7 ± 12.9
Gender (n)	
Male	23
Female	9
Height (cm, mean ± SD)	164.5 ± 8.5
Weight (kg, mean ± SD)	63.8 ± 10.5
Disease duration (month, mean ± SD)	22.9 ± 17.1
H&Y Stage	
1	18
2	9
3	5
Injected dose (MBq, mean ± SD)	428.1 ± 54.3
Injected dose per weight (MBq/kg, mean ± SD)	6.91 ± 1.58
Waiting time (min, mean ± SD)	70.9 ± 9.4

SD, standard deviation

### Subjective assessment of image quality

Subjective image quality was assessed using a 5-point Likert scale across different PET scan durations (ranging from G900 to G30), and the results were listed in Table 2. The results indicated a decline in image quality and lesion conspicuity with reduced scan times. The overall G900, G720, and G600 image quality scores were 5.0 ± 0.0, 5.0 ± 0.0, and 4.9 ± 0.3 points, respectively, and there was no significant difference among these scan duration groups (*P* > 0.05). Notably, there was a significant decrease in these scores at durations shorter than 600 s when compared to G900 images (*P* < 0.05). As the scan duration decreased to 30 s, these scores significantly dropped, with overall image quality reaching as low as 1.5 ± 0.6, image noise 1.5 ± 0.6, and lesion conspicuity 1.4 ± 0.6.

**Table 2:** Subjective image quality assessed with a 5-point Likert scale

	G900	G720	G600	G480	G300	G180	G120	G60	G30
Overall image quality	5.0 ± 0.0	5.0 ± 0.0	4.9 ± 0.3	4.8 ± 0.4*	4.5 ± 0.6*	3.6 ± 0.8*	3.0 ± 0.6*	2.3 ± 0.6*	1.5 ± 0.6*
Image noise	5.0 ± 0.0	4.9 ± 0.2	4.9 ± 0.2	4.8 ± 0.4*	4.3 ± 0.6*	3.5 ± 0.7*	2.9 ± 0.7*	2.3 ± 0.6*	1.5 ± 0.6*
Lesion conspicuity	5.0 ± 0.0	5.0 ± 0.0	4.9 ± 0.2	4.9 ± 0.4	4.5 ± 0.6*	3.6 ± 0.8*	3.1 ± 0.9*	2.1 ± 0.9*	1.4 ± 0.6*

All data are presented as the mean ± standard deviation

* Indicates *P* < 0.05 and G900 images serve as a quality reference

**Fig. 1:**
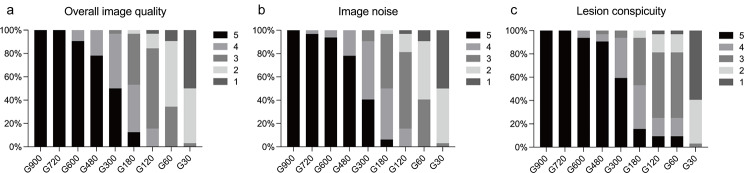
The distribution of subjective image quality scoring among different groups. (a) Overall image quality; (b) Image noise; (c) Lesion conspicuity

As shown in Fig. 1, 100% of the G720 cases and 95% of the G600 cases were evaluated as 5 points when G900 images served as a quality reference, and all the G480 and G300 images were clinically acceptable (≥ 3 points). However, the G180 to G30 images had an increased frequency of score 1 and 2. Similarly, image noise and lesion conspicuity ratings followed this trend. The lesion conspicuity scores were significantly higher in the G900, G720, G600, and G480 than those in the other groups (all *P* < 0.05), while there was no significant decrease in the lesion conspicuity score at durations longer than G300 when comparing to G900 images (all *P* > 0.05).

### Semi-quantitative image analysis

The results of semi-quantitative image analysis are listed in Table 3. Measurements of G900 were served as reference, and a consistent decrease was observed in SUVmean and DAT binding across different brain regions as the scan duration shortened. For example, the SUVmean in the left caudate nucleus decreased from 3.61 ± 1.06 in G900 images to 3.50 ± 1.12 in G30 images. Similarly, DAT binding values in these regions also decreased with shorter scan times. The changes of SUVmean and DAT binding were statistically significant in most cases as indicated by *P* < 0.05. This trend was consistent across various regions, such as the caudate nucleus and putamen. However, there was no statistically significant difference in SUVmean of the occipital cortex among all the groups (*P* > 0.05).

**Table 3:** Semi-quantitative analysis between different acquisition time groups

	G900	G720	G600	G480	G300	G180	G120	G60	G30
**SUVmean**									
CAU_L	3.61 ± 1.06	3.60 ± 1.06	3.59 ± 1.06	3.56 ± 1.07*	3.53 ± 1.08*	3.52 ± 1.07*	3.51 ± 1.08	3.51 ± 1.10	3.50 ± 1.12*
CAU_R	4.10 ± 1.14	4.07 ± 1.14*	4.06 ± 1.13*	4.03 ± 1.13*	4.00 ± 1.11*	3.98 ± 1.11*	3.96 ± 1.10*	3.93 ± 1.10*	3.89 ± 1.11*
PUT_Ant_L	5.18 ± 1.47	5.16 ± 1.46*	5.16 ± 1.46*	5.15 ± 1.44*	5.12 ± 1.41*	5.11 ± 1.39*	5.10 ± 1.39*	5.05 ± 1.35*	5.01 ± 1.34*
PUT_Ant_R	4.78 ± 1.46	4.78 ± 1.47	4.78 ± 1.47	4.77 ± 1.48	4.75 ± 1.48	4.74 ± 1.51	4.74 ± 1.50	4.71 ± 1.48	4.74 ± 1.49
PUT_Post_L	4.05 ± 1.13	4.03 ± 1.12*	4.03 ± 1.11	4.02 ± 1.10*	3.99 ± 1.09*	3.99 ± 1.06*	3.98 ± 1.06*	3.96 ± 1.04	3.94 ± 1.08*
PUT_Post_R	3.88 ± 1.15	3.88 ± 1.18	3.88 ± 1.20	3.87 ± 1.19	3.85 ± 1.17*	3.84 ± 1.17*	3.81 ± 1.17*	3.82 ± 1.15*	3.83 ± 1.16
Ocp	2.14 ± 0.46	2.14 ± 0.46	2.15 ± 0.46	2.15 ± 0.47	2.15 ± 0.47	2.15 ± 0.48	2.15 ± 0.47	2.15 ± 0.48	2.15 ± 0.48
**DAT binding**									
CAU_L	0.69 ± 0.38	0.68 ± 0.38	0.67 ± 0.37*	0.66 ± 0.37*	0.64 ± 0.37*	0.63 ± 0.36*	0.64 ± 0.37*	0.63 ± 0.39*	0.63 ± 0.40*
CAU_R	0.92 ± 0.34	0.91 ± 0.34*	0.90 ± 0.33*	0.89 ± 0.33*	0.87 ± 0.32*	0.86 ± 0.32*	0.85 ± 0.32*	0.83 ± 0.31*	0.82 ± 0.31*
PUT_ Ant_L	1.41 ± 0.43	1.40 ± 0.42*	1.40 ± 0.42*	1.40 ± 0.41*	1.38 ± 0.39*	1.37 ± 0.38*	1.37 ± 0.38*	1.35 ± 0.37*	1.33 ± 0.36
PUT_ Ant_R	1.23 ± 0.46	1.23 ± 0.45	1.22 ± 0.45	1.22 ± 0.46	1.21 ± 0.45*	1.20 ± 0.45*	1.20 ± 0.45*	1.19 ± 0.43*	1.20 ± 0.44
PUT_Post_L	0.89 ± 0.36	0.88 ± 0.35*	0.88 ± 0.34*	0.88 ± 0.34*	0.86 ± 0.32*	0.86 ± 0.31*	0.86 ± 0.31*	0.85 ± 0.30*	0.84 ± 0.29*
PUT_Post_R	0.83 ± 0.44	0.82 ± 0.44	0.82 ± 0.44	0.82 ± 0.44*	0.81 ± 0.42*	0.79 ± 0.41*	0.78 ± 0.41*	0.79 ± 0.40*	0.79 ± 0.39

CAU, caudate nucleus; PUT, putamen; Ocp, occipital cortex; Ant, anterior; Post, posterior; L, left; R, right; DAT, dopamine transporter

All data are presented as the mean ± standard deviation

*Indicates *P* < 0.05, and G900 images serve as a quality reference

**Fig. 2:**
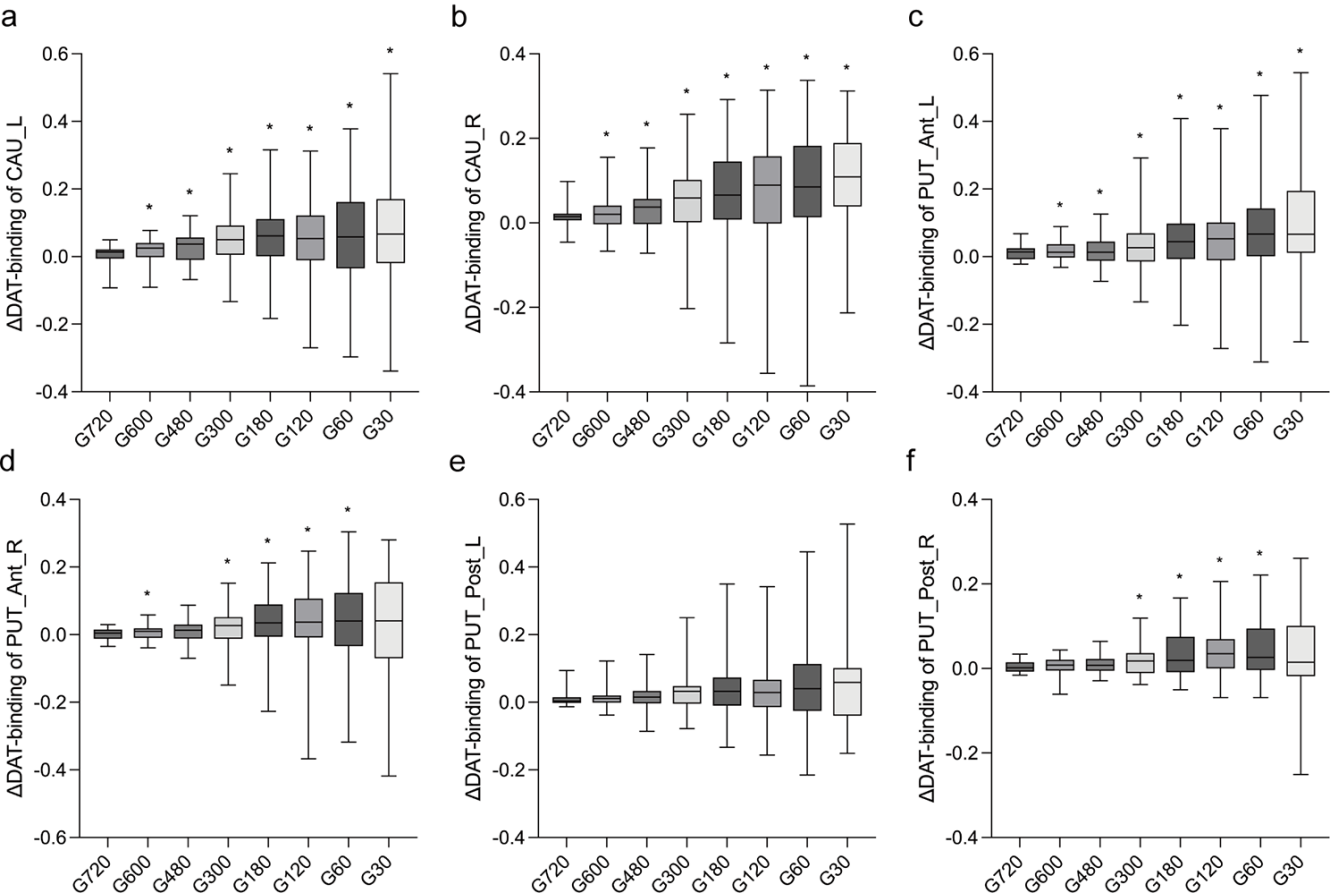
Box plots showing the changes of DAT binding (ΔDAT-binding) of CAU_L (a), CAU_R (b), PUT_Ant_L (c), PUT_Ant_R (d), PUT_Post_L (e) and PUT_Post_R (f) compared with the G900 images (*P < 0.05). ΔDAT-binding was calculated by subtracting DAT binding of G720 to G30 from G900 for each patient. The difference of ΔDAT-binding was analyzed using G720 as reference. CAU, caudate nucleus; PUT, putamen; Ant, anterior; Post, posterior; L, left; R, right

The changes in the DAT binding (ΔDAT-binding) between the full-time reference G900 image and other reconstructed group G720 to G30 images are plotted in Fig. 2. The variability of DAT binding generally increased along with the shortened acquisition time.

### Lesion detectability

The lesion detectability remained 100% on the PET brain images with scan durations longer than 180 s (G900-G180), while it decreased to 96.9% (*n* = 31), 62.5% (*n* = 20), and 28.1% (*n* = 9) in G120, G60, and G30 images, respectively.

## Discussion

PD is one of the most common neurodegenerative diseases, characterized by the loss of dopamine neurons in the substantia nigra and striatum [2, 19, 20]. ^11^C-CFT PET brain imaging, with its specific binding to DAT, plays a vital role in visualizing and assessing the density of presynaptic dopaminergic neurons in the striatum [4]. A notable reduction in ^11^C-CFT uptake in regions like the caudate nucleus and putamen has been observed in PD patients, underscoring its diagnostic significance [7, 8]. Recent consensus and guidelines suggest obtaining ^11^C-CFT PET brain images at least 60 min post-injection for a 15–20 min duration [10]. However, such prolonged scan durations can compromise patient comfort and increase the likelihood of movement-induced artifacts. Conversely, excessively short PET scan times might not allow for optimal tracer detection that is essential for accurate dopaminergic function assessment.

According to previous simulation research, the total-body PET/CT scanner (uEXPLORER) could provide gains of 40-fold sensitivity for total-body imaging compared with the conventional PET/CT with short axial extent. Moreover, the detection performance can still be improved by approximately 4–5 fold for single-organ imaging benefiting from its excellent detector efficiency and timing resolution [11, 13, 21, 22]. Previous studies have demonstrated that the acquisition time can be effectively reduced, and high diagnostic efficacy can be achieved in oncological patients using total-body PET/CT. Notably, recent research utilizing total-body PET/CT for ^11^C-CFT dynamic imaging has provided insights into the real-time internal biodistribution in PD patients [23]. However, there have been few studies about rapid acquisition of ^11^C-CFT PET brain imaging for PD assessment using a total-body PET/CT scanner. Therefore, this study sought to identify the shortest viable PET scan duration that still preserves adequate image quality using ^11^C-CFT, addressing this critical need in PD imaging.

**Fig. 3:**
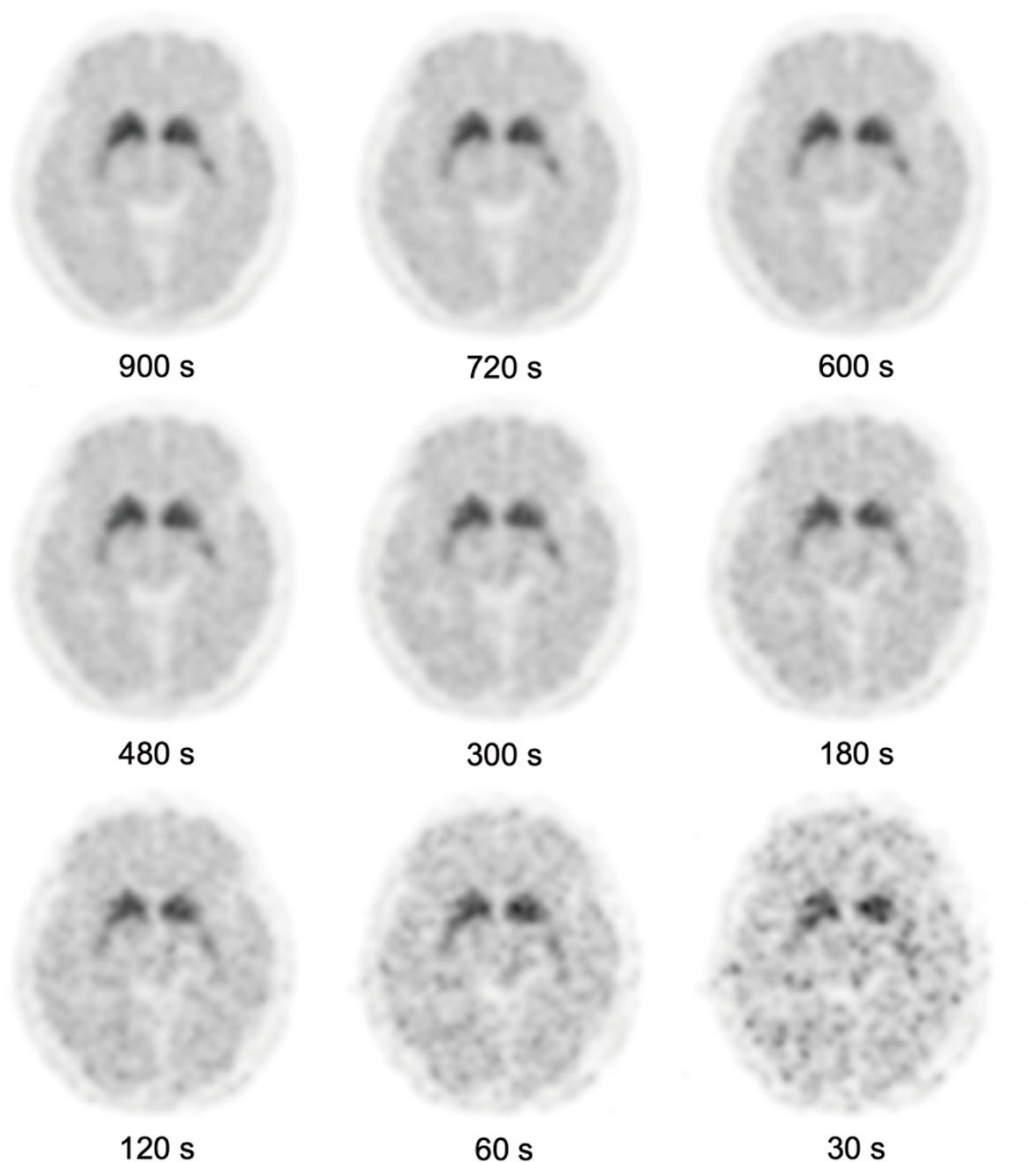
A 54-year-old man with PD underwent ^11^C-CFT PET/CT brain scan. The axial images showed decreased CFT uptake in bilateral putamen. The serial PET brain images were generated by shortening the length of frame duration used for reconstruction. The overall image quality scores of the G900 to G30 images were 5, 5, 5, 4, 4, 3, 3, 2, and 1, respectively

Our findings showed that for the G900, G720, and G600 images, the overall image quality, image noise, and lesion conspicuity scores were consistently high. The scan duration of 600 s sufficiently preserved image quality necessary for accurate assessment of key brain regions like the caudate nucleus and putamen, which are crucial for early detection and disease progression monitoring. Subjective image quality assessments indicated that the G600 image maintained a relatively high overall image quality score (4.9 ± 0.3), with minimal compromise in image noise and lesion conspicuity. Semi-quantitative measurements further corroborated that SUVmean and DAT binding values at G600 of various regions of interest only exhibit minor reductions compared to longer durations, suggesting that the quantitative integrity of the PET images was still preserved at this duration. As shown in Fig. 3, when the scan durations were below 600 s, image noise was significantly enhanced and the boundaries of the basal ganglia structures were gradually blurred, especially the posterior putamen, where CFT uptake showed the most paramount reduction, leading to poor lesion conspicuity. Therefore, this study indicated that the scan duration of 600 s appeared to be a reasonable balance between the need for high-quality imaging and ensuring patient comfort in a clinical setting for PD assessment using ^11^C-CFT total-body PET/CT.

Although previous studies such as those utilizing total-body PET/CT in oncology have explored similar concepts [14–16, 24], our study focusing on ^11^C-CFT in PD fills a specific niche. Differing from the primary concern of lesion detectability in oncological PET imaging, the uniformity and consistency of tracer uptake are crucial in PD assessment. Notably, we observed in this study that although noise affected lesion visualization in areas with reduced CFT uptake, qualitative diagnoses remained largely unaffected due to the inherent “negative imaging” characteristics of CFT PET scans, except in images with excessive noise at lower photon counts (G120-G30). The lesion detectability remained high (100%) until a scan duration of 180 s, but significantly dropped at shorter durations. In particular, the image quality scores of all G300 images were acceptable (≥ 3 points), which was considered to meet the needs of clinical diagnosis.

Moreover, in previous studies investigating low dose of ^18^F-FDG PET, the objective analyses showed pronounced increase in background SUVmax and SD as the acquisition time reduced [14–16]. This increase was explained by noise amplification with reduced scan duration, therefore resulting in a higher maximum pixel value in the background measurement. Our findings demonstrated stability in SUVmean values of the reference region (occipital cortex) across various scan durations in CFT PET, highlighting that SUVmean is a stable parameter for PET assessment and provides a reliable metric in low-count images. Besides, our study demonstrated a trend of decrease in SUVmean and DAT binding of caudate nucleus and putamen as the scan time reduced, which can be attributed to reduced photon detection with shorter scan duration. Besides, a notable observation from our study was the increased change in lesion DAT binding with reduced scan time. This pattern aligned with findings from previous studies and was likely attributable to amplified noise in shorter-duration scans [15]. However, an interesting aspect of our data was the relatively low variability in the DAT binding of the posterior putamen. This observation can be explained by the inherently low DAT binding in this region, a consequence of the pathological characteristics of PD.

The limitations of this study include its small sample size and retrospective design, which may affect the generalizability of the findings. Furthermore, the study’s applicability is primarily limited to the total-body scanner, posing restrictions in broader extrapolation. While reducing PET scan durations in PD assessments could significantly enhance patient comfort and improve the efficiency of medical systems, it is imperative to cautiously evaluate the potential implications on image quality and diagnostic accuracy. A notable concern is that diminished image quality due to shortened scan times might adversely affect the effectiveness of semi-quantitative analysis in assessing the efficacy of therapeutic interventions. Besides, more ^11^C-CFT brain PET data using total-body PET/CT scanner need to be further collected to establish normal reference database for more accurate quantification. These considerations necessitate further investigation in subsequent studies. Advancements in PET imaging technology and the integration of innovative algorithms may provide solutions to these challenges, potentially reducing the required scan time while maintaining high-quality images.

## Conclusion

Sufficient image quality and lesion conspicuity could be achieved at 600-s scan duration for ^11^C-CFT PET brain imaging in PD assessment using a total-body PET/CT scanner, while the image quality of G300 was acceptable for clinical diagnosis. This finding contributes to optimizing PET imaging protocols, which could potentially improve patient comfort and compliance, reduce the likelihood of movement artifacts, and enhance the patient throughput of PET brain imaging.

## Data Availability

The datasets used and/or analyzed during the current study are available from the corresponding author on reasonable request.
